# The Clinical and Economic Impact of Probiotics Consumption on Respiratory Tract Infections: Projections for Canada

**DOI:** 10.1371/journal.pone.0166232

**Published:** 2016-11-10

**Authors:** Irene Lenoir-Wijnkoop, Laetitia Gerlier, Denis Roy, Gregor Reid

**Affiliations:** 1 Department of Pharmaceutical Sciences, Utrecht University, Utrecht, The Netherlands; 2 Director Public Health &Scientific Relations, Danone Company, Paris, France; 3 QuintilesIMS Real-World Evidence, Zaventem, Belgium; 4 Department of Food Sciences, Laval University, Quebec, Canada; 5 Canadian Research and Development Centre for Probiotics, University of Western Ontario, London, Ontario, Canada; Universidade Nova de Lisboa Instituto de Higiene e Medicina Tropical, PORTUGAL

## Abstract

**Introduction:**

There is accumulating evidence supporting the use of probiotics, which are defined as “live micro-organisms which, when administered in adequate amounts, confer a health benefit on the host”, as a preventive measure against respiratory tract infections (RTI). Two recent meta-analyses showed probiotic consumption (daily intake of 10^7^ to 10^10^ CFU in any form for up to 3 months) significantly reduced RTI duration, frequency, antibiotic use and work absenteeism.

**Objectives:**

The aim of this study was to assess the impact of probiotic use in terms of number of RTI episodes and days averted, and the number of antibiotic prescriptions and missed workdays averted, in the general population of Canada. In addition, the corresponding economic impact from both a healthcare payer and a productivity perspective was estimated.

**Methods:**

A microsimulation model was developed to reproduce the Canadian population (sample rate of 1/1000 = 35 540 individuals) employing age and gender. RTI incidence was taken from FluWatch consultation rates for influenza-like illness (2013–14) and StatCan all-cause consultations statistics. The model was calibrated on a 2.1% RTI annual incidence in the general population (5.2 million RTI days) and included known risk factors (smoking status, shared living conditions and vaccination status). RTI-related antibiotic prescriptions and work absenteeism were obtained from the literature.

**Results:**

The results indicate that probiotic use saved 573 000–2.3 million RTI-days, according to the YHEC–Cochrane scenarios respectively. These reductions were associated with an avoidance of 52 000–84 000 antibiotic courses and 330 000–500 000 sick-leave days. A projection of corresponding costs reductions amounted to Can$1.3–8.9 million from the healthcare payer perspective and Can$61.2–99.7 million when adding productivity losses.

**Conclusion:**

The analysis shows that the potential of probiotics to reduce RTI-related events may have a substantial clinical and economic impact in Canada.

## Introduction

Respiratory tract infections (RTI) are highly contagious infections of the sinus, throat, or airways. Typically viral, these self-limiting infections can last up to 2 weeks and vary in severity [1]. Influenza-like-illness (ILI) and influenza are common RTIs, and are defined as acute onset of respiratory symptoms (i.e. cough, sore throat or shortness of breath), accompanied by fever, headache and/or myalgia [1–3]. Cases of laboratory-confirmed influenza virus are termed ‘influenza’ [1].

Due to their high incidence, RTIs carry a heavy burden on society and the healthcare systems. Approximately 5–20% of the population will have at least one RTI annually, resulting in 31.4 million outpatient visits, 3.1 million hospitalized days, and 41 000 deaths each year in the USA [4]. ILI and influenza are estimated to result in 3–5 million illnesses and 250 000–500 000 deaths annually, around the world [5]. In Canada, 14 000–17 000 hospitalizations (8–10% of all hospital admissions) [6] and 3 500 deaths are attributed to influenza each year [7]. The estimated total annual economic burden of RTIs in Canada in 2008, was Can$5.4 billion, representing 2.9% of all healthcare costs [2, 8].

Treatment of RTIs relies mainly on symptom control, however, despite being most commonly of viral etiology, they often lead to the prescription of antibiotics [9, 10]. The use of antiviral agents within 48 hours of illness onset reduces the duration of symptoms by about 1 day; however their effectiveness might be limited by side effects and resistance [11–14]. In the absence of satisfactory treatments, prevention is the cornerstone of influenza management [1, 14]. In addition to limiting contact and frequent hand washing [15], the mainstay of prevention against influenza infection is vaccination [1, 14]. Although influenza is considered to be a vaccine preventable disease, vaccine effectiveness can be limited by mismatches with the circulating viral strains [14] and low uptake in the population [16].

Probiotics, defined as “live micro-organisms which, when administered in adequate amounts, confer a health benefit on the host” [17], are being consumed with increasing frequency over the past ten years. There is accumulating evidence supporting the use of probiotics, both in food products and nutritional supplements, as a preventive measure against RTIs [18–24]. Two recent meta-analyses by the York Health Economics Consortium (YHEC) and the Cochrane Collaboration [18, 20], showed that probiotic consumption reduced RTI duration by 0.8 days [20] and 1.9 days [18] respectively. Moreover, they reduced the incidence of RTIs by 47% [18], the antibiotic prescription rate by 35% [18] and absenteeism by 17% [20]. We hypothesize that there are potential benefits to the Canadian healthcare system associated with these reductions in RTI incidence and duration, which may contribute in lightening the burden of an increasing scarcity of resources.

## Objective

The primary objective of this study was to assess the clinical impact of probiotics use projected to Canada: number of RTI episodes and RTI days averted, number of RTI-related antibiotics prescriptions and missed work days averted. Our secondary objective was to estimate the related economic impact from a healthcare payer perspective and a productivity perspective.

## Methods

An individual-based model was used to perform a health-economic assessment comparing health outcomes and costs with or without probiotics consumption for the population of Canada. Ethics approval and informed consents were not required for this modeling study.

### Model structure

A state-transition microsimulation model, originally applied to the French healthcare setting [25], was adapted to the Canadian population. An important aspect of this adaptation from the French model is the incorporation of the influenza vaccination status of the Canadian population. The model compared the impact of probiotic consumption *vs* no probiotic consumption, using a 1-day cycle, over a time horizon of 365 days, covering the annual surveillance period of flu in Canada from September 2013 to August 2014. The year 2013–2014 was chosen out of the 3 annual surveillance periods from 2012–13 to 2014–15, as the 2013–14 epidemic was of medium intensity [26].

**Fig 1:**
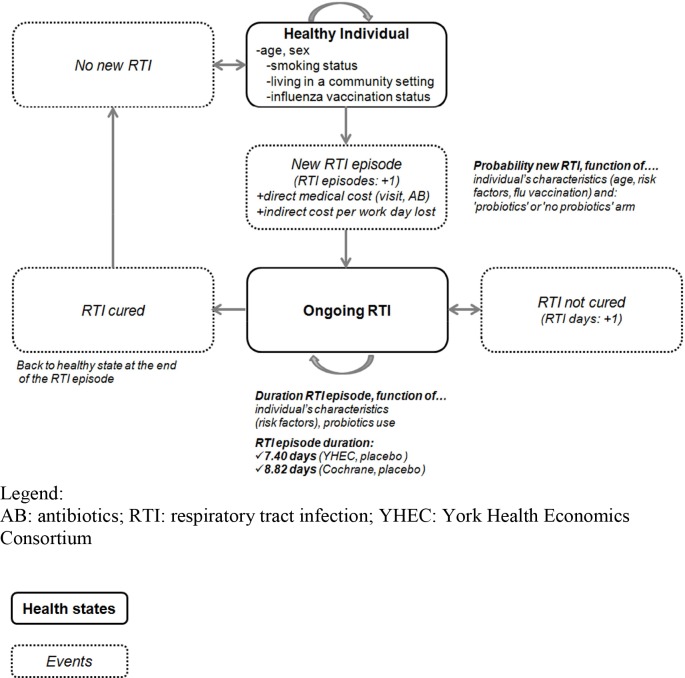
Schematic state-transition model representation.

A sampling rate of 1/1000 virtual healthy individuals, representative of the Canadian population in terms of age and gender were entered into the model. Each subject was concurrently assigned to two arms 'generalized probiotics use' and 'no probiotics use'. Movement from the ‘healthy’ state to an ‘RTI’ state was based on daily age-specific RTI incidence rates adjusted for the following risk factors: smoking status, living in community setting and influenza vaccination status. Individuals remained in a ‘RTI’ state until RTI episode resolution and then returned to the ‘healthy’ state (Fig 1).

Since RTIs as a whole are not subject to herd immunization because of their multi-strain viral origin, the probability of having a new RTI is independent from the number of previous RTIs. Hospitalization and mortality were not incorporated into the model as our scope was restricted to the primary care setting.

The TreeAge Pro 2015 software (TreeAge Software Inc., Williamstown, MA) was employed to conduct the model analyses.

### Model outcomes

The main outcomes of the simulation were the number of RTI events, occurring between September 2013 and August 2014, RTI duration in number of days, number of antibiotic courses prescribed, number of missed work-days due to RTI, direct medical costs (i.e. physician visits, prescribed antibiotics and non-antibiotics drugs) and indirect costs (i.e. productivity loss) from September 2013 to August 2014. The differences between the ‘probiotic’ arm and the ‘no probiotic’ arm in number of events and costs were calculated according to two scenarios based on two meta-analyses, conducted respectively by the YHEC and Cochrane groups [18, 20].

### Perspective

Two perspectives were used in the model: a ‘healthcare payer’ (HCP) perspective, which included RTI-related medical expenses for both public and private payers, and a ‘productivity’ perspective, which focused on productivity losses due to time absent from work.

### Model inputs

A summary of model inputs can be found in Tables 1 and 2.

**Table 1:** Summary of model inputs–Epidemiological parameters, base case Canada.

Model parameters	Value	Reference
Season start-end	Sep 2013-Aug 2014	FluWatch
Time horizon (days)	365	FluWatch
Canada population size	35,154,279	StatCan 2014
**Risk factors**	**% Population**	
**Active smoker**		OFDT 2010
Men, 15-49y	19.5%	StatCan, Tab 105–0501
Men, 50+	9.3%	StatCan, Tab 105–0501
Women, 15-49y	12.0%	StatCan, Tab 105–0501
Women, 50+	7.9%	StatCan, Tab 105–0501
**Passive smoker**	16%	StatCan, Tab 105–0501
**Living in a community setting**		
Pre-school children (0–4)	60%	StatCan
Students (5–15)	100%	UNICEF 2012
Employment rate adults:		
Adults, 15-24y	55.5%	StatCan, Tab 282–0002
Men 25-44y	85.3%	StatCan, Tab 282–0002
Men 45-64y	75.3%	StatCan, Tab 282–0002
Women 25-44y	56.9%	StatCan, Tab 282–0002
Women 45-64y	77.5%	StatCan, Tab 282–0002
Working in open-space offices	70.0%	IFMA (US)
Adults in retirement home, 65-74y	11.0%	StatCan, GSS 2011
Adults in retirement home, 75-84y	35.0%	StatCan, GSS 2011
Adults in retirement home, 85+	55.0%	StatCan, GSS 2011
**Influenza vaccination coverage**		
Children, 0-12y	23%	StatCan
Children, 12-17y	23%	StatCan
Adults, 18-34y	17%	StatCan
Adults, 35-44y	22%	StatCan
Adults, 45-54y	25%	StatCan
Adults, 55-64y	39%	StatCan
Adults, 65y+	64%	StatCan
**Use of probiotics in Canada**		
Overall	Heavy/regular users: 34%/10.7%	IPSOS survey
Male	Regular/heavy users: 23%/7.1%	US survey
Female	Regular/heavy users: 45%/14.2%	US survey
**Steps to RTI incidence estimation**		
**Number of all-cause MD visits:**		
≥ 1 all-cause physician visits, age 12+	N = 23,263,508 (759.69 /1000 persons)	StatCan
≥ 1 all-cause physician visits, all ages	N = 26,936,522 (766.24 /1000 persons)	StatCan + assumption*
	**Total ILI Consultations (ILI consultation rate)**	
**Total ILI consultations 2012–13**	561,771 (1,664 /100,000 persons)	Calculation (FluWatch + StatCan)
**Total ILI consultations 2013–14 (base case)**	735,967 (2,094 /100,000 persons)	Calculation (FluWatch + StatCan)
**Total ILI consultations 2014–15**	789,710 (2,222 /100,000 persons)	Calculation (FluWatch + StatCan)
**RTI duration**	**Duration (days)**	
Without probiotics (placebo)	YHEC: 7.40/Cochrane: 8.82	King et al 2014, Hao 2015
**Impact of risk factors on RTI**		
Active smoker	On RTI incidence: NA/On RTI duration : +4.5% vs. no smokers	Bensenor 2001
Passive smoker	On RTI incidence: RR = 1.15 vs. no smokers/On RTI duration: +16.8% vs. no smokers	Bensenor 2001
Living in a community setting:		
Day care (e.g. school) vs. home care	On RTI incidence: RR = 1.22/On RTI duration : NA	Louhiala 1995
Shared office vs. alone	On RTI incidence: RR = 1.07#/On RTI duration : NA	Jaakkola 1995
**Impact of using probiotics**		
YHEC	On RTI incidence: NA/On RTI duration: -0.77 days vs. pbo [-0.04;-1.50] /On antibiotics use : NA/On work absenteeism : -0.17 SMD [-0.31 ;-0.03]	King et al 2014
Cochrane	On RTI incidence: RR = 0.70 vs. pbo [0.50;0.84]##/On RTI duration: -1.89 days vs. pbo [-2.03;-1.75]/On antibiotics use : RR = 0.65 vs. pbo [0.45;0.94]/On work absenteeism : NA	Hao et al 2015

IFMA: International Facility Management Association; MD: Medical doctor; NA: not applicable; pbo: placebo; SMD: standardized mean difference

* Assumes same visit rate in <12 years and 65+ years

#OR = 1.64

##OR = 0.53 [0.37;0.76]. Conversions into RR using exact numbers of events and sample sizes

**Table 2:** Summary of model inputs–Resource utilization and costs parameters, base case Canada (2015 costs).

**Direct cost parameters**	**% using the resource**	**Number, mean**	**Unit cost /(% paid by public payer)**	**Reference**
GP visits in case of RTI	100%	1 visit	Can$32.00 (100%)	Family Health Online Canada
Analgesic/anti-pyretic in case of RTI	90%	1 pack for 7 days	Can$6.29 (0%)	Well.ca
Antibiotics course, in case of RTI	26.1%	1 course of 10 days	Can$25.00 (30%)	Kwong et al 2009, Canadian RX atlas
**Indirect cost parameters**	**% missing days**	**Number of missed days, mean**	**Cost per day lost/(Employer cost)**	**Reference**
employee with RTI	42.0%	1.7 days	Can$181.61	Palmer et al 2010, GDP per capita
sick children with RTI	18.0%	0.5 days	Can$181.61	Palmer et al 2010, GDP per capita

Can$: Canadian Dollar

GDP: gross domestic product

GP: general practitioner

ILI: influenza-like illness

RTI: respiratory tract infection

#### Demographic data

In 2014, the total Canadian population was 35 540 000 individuals, and was reported by gender and 5-year age increments [27].

#### RTI incidence

The type of RTIs included in this model were ILIs, as reported by FluWatch [28], Canada's national surveillance system that monitors the spread of epidemics. The FluWatch program collects data from a network of labs, hospitals, doctor's offices and provincial and territorial ministries of health and includes only patients who have consulted with a physician for an ILI, and therefore, represents a subset of all patients with ILIs in Canada [29].

ILIs as defined by FluWatch (“acute onset of respiratory illness with fever and cough and with one or more of the following—sore throat, arthralgia, myalgia, or prostration which is likely due to influenza”) [29] are included in the definitions of RTIs symptoms used in the Cochrane (“common cold and inflammation of the trachea and larynx, with symptoms including fever, cough, pain and headaches”) [18] and YHEC (“colds or influenza-like symptoms”) [20] meta analyses. Both meta-analyses included studies of patients with acute RTIs similarly defined, however, they also included patients with common cold, thus, our criteria were more restrictive.

In the absence of published Canadian absolute ILI incidence, the incidence of RTIs was calculated and derived from the weekly ILI consultation rate per 1000 physician visits in the general Canadian population [26], by age group, for the years 2012–13, 2013–14 and 2014–15 (Table 1). The number of all-cause physician visits was estimated from the Statistics Canada website (number of individuals with ≥ 1 physician contact per year, per age group [30]) assuming a single contact per year per consulting individual. For model purposes, the all-cause consultations were assumed to be uniformly distributed over the year and the consultation rate among 0–12 year age group was assumed equal to that among ≥ 65 year age group [31].

#### Effect of probiotics on RTI

Two scenario analyses were conducted independently, using the results from the two meta-analyses. Both scenarios are thus based on different assumptions. In the first scenario, using the data reported by YHEC [20], the estimated impact of probiotics was based on an average RTI duration of 7.40 days and a reduction of duration of -0.77 days [-1.50, -0.04]. The second scenario, using the Cochrane data [18] was based on an average RTI duration of 8.82 days, a reduction of duration of 1.89 days [-2.03, -1.75] and a reduced risk of an RTI incidence of 0.70 among non-vaccinated individuals only (in line with the inclusion criteria of this meta-analysis). Both scenarios additionally used a reduced risk of receiving an antibiotic prescription per RTI episode of 0.65, among non-vaccinated individuals only, and reduced absenteeism of 0.87 and 0.26 days among adults and children, respectively.

Currently, an estimated 12% of the Canadian population regularly consumes probiotics. This consumption rate was used to adjust the RTI incidence and duration per RTI episode of the general population in the model. The percentage of Canadians currently consuming probiotics was estimated from two sources: a recent survey on health product consumption in the overall Canadian population and a US study reporting the ratio of men and women using probiotics, resulting in an estimated 8% of men and 15% of women (and 12% overall) as regular consumers of probiotics [32, 33].

#### Resource use and costs

For each RTI consultation, the cost of one visit to the family physician was attributed at a unit cost of Can$32 [34]. It was assumed that 90% of the consulting RTI patients take over-the-counter analgesic or anti-pyretic medications, at a unit cost of Can$6.29 for 7 days of treatment with ibuprofen [35]. In Canada, an estimated 26.1% of consulting RTI patients are prescribed antibiotics for RTI at a unit cost of Can$25.00 per antibiotic course [36]. In terms of reimbursement from public HCP sources, the visit cost is fully covered, while the public insurers covered an estimated 30% of antibiotic prescriptions in 2012/2013 [37]. Pain/fever medications are assumed to be self-medication, out-of-pocket expenses for the patient. Resource costs are presented in Table 2.

The cost of probiotics was not incorporated into this projection. Firstly, the study aimed at assessing the benefits of routine probiotics consumption. In addition, probiotic products, whether they are part of daily food consumption or purchased in the form of nutritional supplements, are not reimbursed by the healthcare system, and thus fall beyond the scope of the study perspective (HCP and productivity losses). This point is further addressed in the discussion. The heterogeneity of the types of commercialized probiotics and the lack of available data on consumer habits, make it difficult to quantify the type, amount and cost of probiotics consumed.

Indirect costs included the productivity losses caused by the working days lost due to RTI. In the absence of Canadian specific data, estimates were derived from a US study which reported the number of missed working days caused by RTI, among employed adults for their own illness and illness of their children [38]. The cost of 1 day of lost productivity was estimated at Can$182, based on the Canadian gross domestic product (GDP) per capita divided by 250 working days per year.

All costs were obtained from Canadian sources and are expressed in 2016 Canadian dollars.

#### RTI risk factors

In the model, RTI incidence and/or duration were adjusted for known risk factors of smoking status, living in a community setting status (i.e. child attending day care or school, employed adults working in open offices, or elderly in a retirement home) and influenza vaccination status. The risk factor probabilities used in the model and their impact on RTI are reported in Table 1

#### Smoking status

A study based on randomized controlled trial data [39] showed that active smokers were more likely to report a prolonged RTI episode (> 7 days vs. 1–3 days) compared to never smokers: light smokers (< 25 cigarettes a day) had a relative risk of 1.62 [1.40, 1.87] and heavy smokers (≥ 25 cigarette a day), had a risk of 2.63 [2.02, 3.44] [39]. Non-smokers exposed to second hand cigarette smoke also reported a longer duration of upper RTI (RR = 1.12 [0.99, 1.27]) vs. never smokers. From these RR, the average RTI duration was estimated to be 16.8% [9.1%, 25.2%] longer for active smokers and 4.5% [0.1%, 8.9%] longer for passive smokers, compared to never smokers (Table 1). In addition, RTI incidence was assumed to be higher among passive smokers (RR = 1.15 [1.05, 1.26]) *vs* never smokers (Table 1).

The proportion of active [40] and passive smokers [41] in Canada, by age group and sex, was obtained from Statistics Canada.

#### Living in a community setting

The relative risk of an RTI event among children attending day care *vs* home care (RR = 1.22 [1.13, 1.31]) [42] was applied to children aged 0–4 years attending day care and children aged 5–15 years attending school *vs* children staying at home (Table 1). For employed adults aged 16–64 years old working in a shared office and elderly people aged above 65 living in a retirement home, an increased risk of RTI (RR = 1.07 [1.01;1.13]) was applied [43]. The proportion of Canadians living in a community setting, by age group, was obtained from Statistics Canada.

#### Influenza vaccination

Two recent systematic reviews on influenza vaccination reported decreased risks of ILI among vaccinated adults (RR = 0.83 [0.78, 0.87]) [44] and children (RR = 0.64 [0.54, 0.76] [45], which were applied in the model given that our RTI events are matching the ILI definition (Table 1). Since vaccination against influenza is recommended for everyone 6 months of age and older in Canada [46], the probiotic effects estimated from the Cochrane meta-analysis were not applied to individuals in our model who were vaccinated against influenza, to keep in line with the inclusion criteria of the Cochrane meta-analysis; under the YHEC scenario RTI rates were adjusted for vaccination status. The proportion of Canadians who were vaccinated for the 2013–14 season were obtained from the 2015 Canadian Community Health Survey [47].

### Analyses

The model population was analyzed using SAS software (SAS Institute, Cary, North Carolina) and results are presented for 35 540 individuals (model sampling rate 1/1000). One-way sensitivity analyses to assess uncertainty around the results were performed on the preceding (2012–13) and following (2014–15) influenza seasons, which had slightly lower and higher RTI rates, respectively, and across the lower and upper 95% confidence limits for reduced duration of RTI and reduced incidence of RTI.

## Results

**Fig 2:**
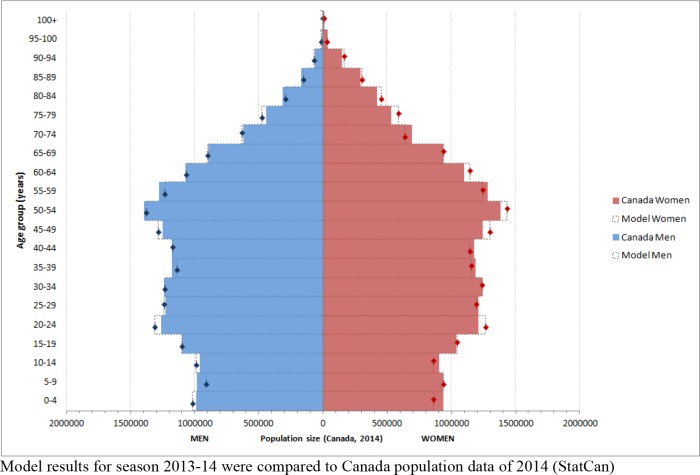
Canada population structure by age and gender, from national statistics (colored bars) vs. simulated population (dotted line bars).

A sampling rate of 1/1000, or 35 540 simulated individuals, reproduced the Canadian population structure in terms of age and gender (Fig 2), with an error rate less than 5% between expected population size and modeled population size.

**Fig 3:**
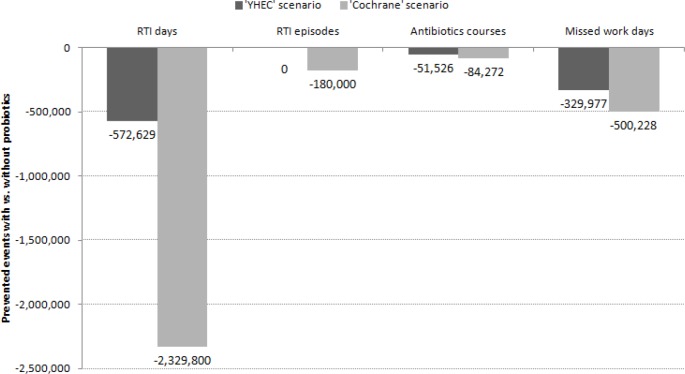
Prevented RTI-related events with vs. without probiotics according to two scenarios.

Under the YHEC scenario, which focused on the probiotic effect on RTI duration, projected to the Canadian population over a one year period (Sept 2013 –Aug 2014), probiotic consumption would avert 572 629 days of RTI illness (10.4% reduction), 51 526 antibiotic prescriptions for RTI (26.4% reduction) and 329 977 days of missed work (35.9% reduction), compared to no probiotic consumption (Fig 3). Under the Cochrane scenario, which focused on the effect of probiotics on reducing both RTI incidence and duration, over the same time period and projected to the Canadian population, probiotics consumption would avert 2 329 800 RTI days (35.3% reduction), 180 000 RTI episodes (23.9% reduction), almost 84 272 antibiotics prescriptions for RTI (42.8% reduction) and 500 228 missed work days (51.3% reduction), compared to no probiotic consumption (Fig 3).

**Fig 4:**
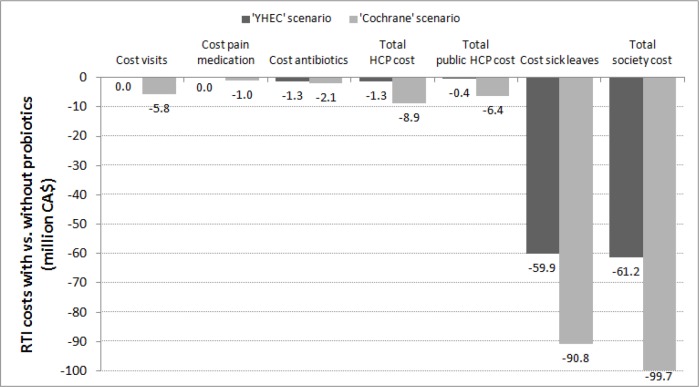
Savings with vs. without probiotics according to two scenarios.

In terms of economic impact, the cost reduction associated with the averted RTI events amount to Can$1.29 million from the HCP perspective and Can$61.22 million when taking productivity losses into account (-30.6%), based on YHEC scenario (Fig 4).

In the Cochrane scenario, the economic impact of averted RTI events was estimated at Can$8.89 million from the HCP perspective and Can$99.77 million when productivity losses are included (Fig 4).

A higher relative benefit of probiotic consumption on the reduction of RTI duration was observed among children < 10 years old, on individuals living in a community setting and on those who were not vaccinated against influenza. Children < 10 years old represented 10.5% of the population (N = 3 721 000) but accounted for 19.5% of the potentially averted RTI days (-111 650 days), thus, this young age group shows a higher incremental benefit than other age groups. Individuals working or living in a community setting accounted for 50.7% (N = 18 000 000) of the total population but accounted for 56.8% (-325 459 days) of the total RTI-days saved with probiotics consumption. Non-vaccinated individuals represented 69.4% of the total Canadian population in 2014, these individuals accounted for 74.5% of the total RTI-days potentially averted in the general population under the YHEC scenario, meaning that even vaccinated individuals can have some benefits of probiotics.

The results of the sensitivity analyses showed that the model results are robust against varying RTI rates from the preceding (2012–13) and following (2014–15) seasons, and the lower and upper 95% confidence limits of RTI duration and RTI incidence.

The impact of probiotic consumption on RTI days based on 2012–13 and 2014–15 seasons did not differ substantially from the base case results. Averted RTI days ranged from 465 080 to 582 890 in the YHEC scenario (corresponding HCP costs of averted RTI events Can$1.07–1.33 million and Can$50.63–63.76 million when including productivity losses), and from 1.62 to 2.42 million days in the Cochrane scenario (corresponding HCP costs of averted RTI events Can$ 5.87–8.99 million and Can$70.44–103.17 with productivity losses).

Applying the lower and upper 95% confidence limits of the reduction of RTI duration from the YHEC meta-analysis (-0.04 to -1.50 days per episode), the potentially averted RTI days in the probiotics arm varied between 29 720 and 1.12 million days; from the Cochrane meta-analysis (-2.03 to -1.75 days per episode), the potentially averted RTI days in the probiotics arm varied between 2.27 and 2.38 million.

When testing the 95% CI around the reduction of RTI incidence from the Cochrane meta-analysis (RR from 0.50 to 0.84), the averted RTI days in the probiotics arm varied between 1.67 and 2.92 million days.

## Discussion

The microsimulation described here estimates the potential clinical and economic benefits of probiotic consumption on RTIs in Canada, under two distinct scenarios derived from two recent meta-analyses [18, 20]. The model was anchored on the Canadian population structure and RTI incidence data were applied. Projecting the clinical benefits onto the Canadian population demonstrates that probiotic consumption has the potential to save 180 000 RTI episodes and 500 000–2.3 million RTI-days with an associated avoidance of 50 000–85 000 antibiotic courses and 300 000–500 000 work absenteeism days. These averted RTI events, when translated into averted costs for the HCP, would represent Can$1.3–8.9 million and up to Can$61.2–99.7 million when including the averted costs of productivity losses.

Our findings are consistent with a similar analysis conducted on the French population, which showed that population level probiotic consumption in France would potentially save 2.4–6.6 million RTI sick days, 291 000–473 000 antibiotic courses and 581 000–1.5 million work absenteeism days. The economic impact of preventive probiotic use was estimated to be €14.6 - €37.7 million to the French National Health Care System [25]. The main reason for a higher probiotic impact in the French analysis is that the RTI definition was encompassing not only ILI but also common colds.

Data on common colds were indeed not available for the Canadian population, therefore only ILIs were included in the model, resulting in a more restrictive inclusion. As well, unlike the French model, the impact of confounding due to vaccination against influenza was decreased in the Canadian model by including the vaccination status of the population. Both of these factors led to a more conservative model.

As in the French model, the Canadian model shows a higher incremental benefit of probiotic consumption among children < 10 years old and individuals living in a community setting. This is likely due to a higher incidence of ILIs among children [48] and the ease of transmission among individuals who go to school and work in close proximity to others [42, 43]. The Canadian model also shows higher benefit among people not vaccinated against influenza.

In both French and Canadian analyses, the acquisition cost of probiotics was not included since probiotics are purchased on a voluntary basis by the families without any subsidy or reimbursement, independent of their health status. As such, the cost of probiotics is not part of the HCP perspective adopted in our study. The particularity of probiotics, and healthy/functional food in general, compared to a standard health intervention is that individuals can decide to acquire probiotics for several reasons (taste/preference over non-fermented dairy products, healthier diet purpose,…) and the potential RTI prevention properties might only be part of it. From the large Canadian survey on healthy food [32], the average household budget for such products in heavy vs light users is Can$175 vs. Can$128 per week i.e. an incremental weekly expense of Can$47 for heavy users. This might represent the willingness to pay (WTP) of Canadian households for healthy food including probiotics. In comparison, the cost of probiotics in France per household was estimated between Can$182 and Can$484 for a period of 7 months i.e. Can$7–17 per week. This suggests that the acquisition cost of probiotics is largely inferior to the WTP for healthy food, and this without any incentives (aside private advertising). Other out-of-pockets expenses that were not included in this model due to lack of data were over-the-counter (OTC) medication and costs related to informal care for a sick parent or child. The above mentioned costs would be part of a so-called ‘society’ perspective, along with any Government expenses on campaigns or advertisements to, for example, encourage healthy lifestyle choices in the population. However, this fell beyond the scope of our analysis.

Importantly, the current incidence data were representative of individuals consulting a GP for their RTI. They represent only a very small proportion of RTI sufferers, and therefore, the real savings may be higher than reported.

The role of functional foods is increasingly being recognized as important, by not only public health departments, but also by payers and policymakers [49]. Epidemiological studies have established the clinical benefits of nutrition and functional foods on disease, including the use of probiotics to prevent diseases [50]. Several meta-analyses of randomized controlled trials have shown a benefit of probiotic interventions in various therapeutic areas including neonatology [51], gastroenterology [52], cardiovascular risk factors [53], urinary [54] and respiratory tract [18, 20]. Along with the clinical benefits, functional foods have the potential to impact healthcare costs. In the current context of competing healthcare dollars, with the challenge of allocating limited funds to an extensive list of needs, functional food–including probiotics- offers an attractive population based strategy for improving health. The emerging discipline of nutrition economics [55], to which this study contributes, will help decision makers to evaluate the relevance of assessing the economic impact of nutrition [49].

This analysis shows that increasing probiotic consumption is likely to have substantial positive consequences, not only on the healthcare system, but also on work absenteeism of sick employees per se as well as those absent because of their children with respiratory illness. This is meaningful, as approximately one third of employees working in an open office plan confirm their working environment puts them at increased risk of illness due to the close and open contact with colleagues [56]. The impact of RTI on work presenteeism (reduced on-the-job productivity due to RTI symptoms) could be another field of research to cover.

## Limitations

Our research is subject to the limitations inherent to all modelization work, and uncertainty around model inputs in particular. First of all, both meta-analyses highlighted important heterogeneity in the included studies. In addition, the meta-analyses of Cochrane are more cautious with regards to the results, qualifying the evidence as “low quality” while YHEC concludes that their results are based on “a number of good quality RCTs”. In our analysis, we decided to show both scenarios to cover both more optimistic effects based on ‘low quality evidence’ (Cochrane) and more conservative effects based on a higher quality of evidence (YHEC). We acknowledge that the evidence around the preventive effects of probiotics is deemed preliminary by a number of scientists, in view of contradictory results: non-conclusive subgroup analyses by age group (YHEC) or efficacy not sufficiently ascertained according to Caffarelli et al [57], while two other meta-analyses including moderate quality [58] to high-quality [59] studies confirm the positive effect of probiotics on RTI incidence and symptoms. Despite practical or ethical challenges, it is expected that the quality of RCTs conducted in the nutrition area will substantially improve in the near future [60, 61]. Another limitation of our work concerns the RTI definitions and labels used in the meta-analyses, which are approximations of the ILI infections as defined by FluWatch; the overlap was deemed acceptable though.

Furthermore seasonality data of all-cause visits was not available from Statistics Canada, therefore an average number of weekly all-cause visits was assumed throughout the year, based on the rate of individuals who had at least one visit to a health care professional in the past 12 months. This is most likely a very conservative assumption as we counted a single all-cause visit per consulting patient (in the US, the average is around 3 visits per person-year). And lastly, because Statistics Canada does not report the all-cause consultation rate on the population < 12 year old, we assumed this age group was equal to the ≥ 65 year age group in terms of visit rate, as was observed in the US statistics [62]. These modifications can artificially inflate or deflate the RTI incidence at various points during the year. These limitations may have consequences on the model outcomes when examined by subgroup, as we assumed that risk factor prevalence and effects were independent. The overall impact on the Canadian data is not considered to be high, as the model remained correctly anchored on national statistics, for 3 years in a row. In terms of economic results, there is some uncertainty around the duration of ILI symptoms potentially requiring medications; package size of analgesics/antipyretics was estimated based on the duration of RTI episodes in the placebo groups of the meta-analyses (7–8 days) while the cost of an antibiotic course was directly provided by a Canadian health information website.

## Conclusion

This study shows the potential for a substantial reduction of RTI events and related HCP costs and productivity losses if probiotics would be consumed routinely at a population level in Canada. The model projects a higher relative benefit of probiotic consumption among children < 10 years old, individuals living in a community setting and those not vaccinated against influenza. Further good quality, prospective research on probiotics effectiveness is required to refine our preliminary projections.

## Supporting Information

S1 Table Model inputs and incidence source data. (XLSM)
